# Quasielastic Light Scattering in the Broadband Brillouin Spectra of Relaxor Ferroelectric PbMg_1/3_Nb_2/3_O_3_

**DOI:** 10.3390/ma16010346

**Published:** 2022-12-30

**Authors:** Nikita K. Derets, Alexander I. Fedoseev, Jae-Hyeon Ko, Seiji Kojima, Sergey G. Lushnikov

**Affiliations:** 1 Division of Physics of Dielectrics and Semiconductors, Ioffe Institute, Politekhnicheskaya 26, 194021 St. Petersburg, Russia; 2 School of Nano Convergence Technology, Nano Convergence Technology Center, Hallym University, 1 Hallymdaehakgil, Chuncheon 24252, Gangwondo, Republic of Korea; 3 Division of Materials Science, University of Tsukuba, Tsukuba 305-8573, Ibaraki, Japan

**Keywords:** Brillouin scattering, relaxations, quasielastic light scattering, relaxors ferroelectrics

## Abstract

In this paper, the behavior of quasielastic light scattering (QELS) in a PbMg_1/3_Nb_2/3_O_3_ (PMN) crystal under broadband Brillouin light scattering in a temperature range from 750 K to 80 K was studied. It was shown that QELS consists of two components: narrow (0.9 GHz to 11 GHz) and wide (80 GHz to 600 GHz). The dependencies of the intensity, *I*, of these components on the frequency, *ν*, are well described by the power law *I ~ eν^α^*, with different α, and are determined by the distribution of the relaxation times. The analysis of the Brillouin spectra showed that the behavior of the relaxation time of both the components of QELS with temperature change is well described by the Arrhenius law. Additionally, in the vicinity of the intermediate temperature T* ≈ 380 K, a critical relaxation time behavior for the narrow component of QELS was detected. In the vicinity of the same temperature, a maximum in the integral intensity of both the components of QELS was observed, which is adjacent to another maximum in the region of the Vogel–Fulcher temperature *T*_VF_ ≈ 250 K corresponding to the transformation of the crystal to a nonergodic state.

## 1. Introduction

Quasielastic light scattering (QELS) is an intriguing subject in the physics of the vibrational spectrum of dielectric crystals. This phenomenon is observed in many compounds during phase transitions or transformations, which are studied by optical spectroscopy [1]. The nature of this contribution to elastic scattering at an unshifted frequency in the spectra of inelastic light scattering has been studied in detail. Several mechanisms are described that lead to the appearance of QELS in light-scattering spectra, including the influence of defects, entropy, fluctuation, and others, which differ in their dependence of the QELS width on the wave vector, the behavior of the intensity, the width itself, and the region of existence, with the change in the external thermodynamic parameters (temperature, pressure) [1]. The existence of QELS has also been observed in disordered structures, such as glasses, supercooled liquids, etc. [2,3,4]. In recent years, particular interest in this phenomenon has arisen in the study of the vibrational spectrum of partially disordered crystals and primarily in relaxor ferroelectrics with a perovskite structure. This is due to the fact that the existence of QELS in relaxor ferroelectrics was associated with polar nanoregions (PNRs), which dramatically change the dynamics of the crystal lattice of perovskite ferroelectrics. Indeed, in relaxor ferroelectrics with the general formula $A{B^{\prime }}_{1-x}{B^{\prime \prime }}_{x}O_{3}$, a frequency-dependent anomaly of the dielectric response is observed, which is “stretched” in temperature by hundreds of degrees. This anomaly is not associated with a structural phase transition. Anomalies in the dielectric response correspond to frequency-dependent anomalies in the speed of sound, damping, and several other physical properties [5,6]. For the first time, polarized QELS in relaxors was observed in a PbMg_1/3_Nb_2/3_O_3_ (PMN) crystal in the Raman scattering spectra [7] within a temperature region where an anomalous dielectric response was observed, called the diffuse phase transition region. Later, it became clear that QELS is a characteristic feature of the low-frequency vibrational spectrum of relaxors and is observed in experiments using optical spectroscopy [8,9]. Experimental studies have shown that QELS is observed in a wide frequency range (more than three orders of magnitude in frequency) and has a complex temperature dependence [10,11,12,13]. At the moment, it is assumed that its appearance is associated with the appearance of PNRs (or polar clusters) in the vicinity of the Burns temperature (T_B_). A description of various ideas about PNRs can be found in a number of papers on this topic [14,15,16]. The behavior of QELS with temperature change is determined by the dynamics of these nanoregions [8]. In the current approach describing the dynamics of PNRs, there are two main mechanisms that contribute to the dielectric dispersion and, accordingly, to the anomaly of the dielectric response of relaxors. The first mechanism is related to the dynamical flipping of the cluster polarization. This mechanism determines mainly the high-frequency contribution to the dispersion of the dielectric response. Below the freezing temperature, *T*_VF_, the clusters freeze out and the flipping of the polarization disappears. The second mechanism is determined by the vibration (breathing) of the polar cluster boundaries. Both of these mechanisms determine a broad distribution function for relaxation time, which expands with decreasing temperature. It is the vibrations of the walls of the polar nanoclusters that determine the dispersion of the dielectric response at *T* < *T*_VF_ [9].

Studies into QELS in relaxors have shown that it is polarized and can be complex, i.e., it can consist of several contributions, as was observed for Na_1/2_Bi_1/2_TiO_3_ (NBT) [17]. Studies of the polarized spectra of Brillouin and Raman scattering in PMN-56%PT made it possible to relate the QELS component observed in the vertical–vertical (VV) polarization configuration to the longitudinal component of polarization fluctuations and that observed in a vertical–horizontal (VH) polarization configuration to the transverse component of polarization fluctuations [12,13]. An analysis of the intensity distribution of QELS in PMN crystals in the range of three orders of magnitude of frequency allowed the authors [11] to show its selfsimilarity. The intensity distribution is described as a power-law spectrum using the expression $I∼\nu^{\alpha }$, where *I*, *ν,* and *α* are the intensity, frequency, and power exponent, respectively. The power-law spectrum implies a Lorentzian superposition (Debye relaxators) with different half-widths, that is, with different relaxation times, defined as the reciprocal of the Lorentzian half-width. Thus, in the frequency range under consideration, we have the power-law distribution for relaxation time [11]. Another consequence of such a description of QELS is the conclusion of the authors from [11] on the possible use of the fractal approach to describe the dynamics of the PNRs of a PMN crystal. This conclusion is consistent with the results of the analysis of the generalized density of states function [18] and the behavior of heat capacity [19] in PMN obtained within the framework of the fractal approach. Recent studies on the spatial organization of nanoregions in relaxors [16] support the assumption that fractals exist in PMN crystals. A detailed analysis of dielectric dispersion over a wide frequency range based on infrared reflectivity (IR) and terahertz time-domain spectra combined with high -frequency dielectric data above the megahertz range did not support the picture of fractal dynamics proposed in [20]. Additional studies into QELS in PMN crystals have not been carried out ever since. This motivated us to investigate QELS using Brillouin light scattering in a PMN crystal over a wide range of frequencies and temperatures.

PMN is a model for studying the properties of relaxors. For PMN, the anomaly in the dielectric response in the vicinity of maximum temperature, *T*_m_ ~ 270 K, at a frequency of 10 kHz is not associated with a macroscopic change in symmetry, which remains cubic (space group $Pm\bar{3}m$) down to helium temperatures [21]. The lattice dynamics of the PMN crystal exhibit complex behavior in this case. In the vicinity of 900 K, the appearance of a polarized Raman spectrum of the first order is observed [22]. This effect is called a dynamic phase transition and is associated by the authors (in a number of works) with the nonequivalence of oxygen octahedra at B′ and B″ cations, which begin to manifest themselves at these temperatures [22]. A further decrease in temperature leads to a deviation in refractive index from its high-temperature linearity, which is due to the formation of PNRs (*T*_B_ ≈ 640 K) [23]. In the vicinity of this temperature, quasielastic scattering also occurs in the spectra of inelastic neutron scattering [5]. Its intensity increases with decreasing temperature and reaches a maximum simultaneously with permittivity (at the GHz frequency) in the vicinity of the intermediate temperature, T* ~ 380 K [24]. The anomalies in several of the physical properties of PMN are associated with this temperature [25], including a sharp increase in the intensity of the elastic component in inelastic neutron scattering [24]. The observed features in this temperature range are associated with a transition from a cubic isotropic to a cubic anisotropic state [24] or with the formation of static nanoregions [9,14,25]. Another characteristic point in the discussed pattern of phase transformations in PMN is the temperature of the ferroelectric phase transition induced by an applied electric field in the vicinity of 210 K [5]. In the absence of an electric field, a low-temperature region is isolated at *T*_VF_ < 250 K, where the dynamics of the PNRs “freeze” and a nonergodic state appears [14].

The dynamics of the PNRs in PMN in a wide frequency range have been studied in sufficient detail using dielectric, IR, and terahertz time-domain spectroscopy [5,9,20]. Broadband Brillouin scattering is a complementary method for studying the dynamics of PNRs in a frequency range from 10^9^ to 10^12^ GHz, which allows one to study the relaxation mechanisms in the gigahertz frequency domain. This motivated us to investigate the temperature behavior of the low-frequency region of the vibrational spectrum of a PMN crystal using Brillouin light scattering in a frequency range from 0.6 GHz to 800 GHz within the temperature range, which includes the regions of appearance, transformation, and freezing of the PNRs.

## 2. Materials and Methods

In this work, the low-frequency region of the vibrational spectrum of a PMN crystal was studied in detail by means of Brillouin light scattering. The PMN crystal was grown by the Czochralski method at the SI Vavilov State Optical Institute (JSC SI Vavilov State Optical Institute, St. Petersburg, Russia) in the form of a large boule with a diameter of about 30 mm and a length of more than 60 mm under the direction of [100]. Part of the boule was used to prepare large samples (up to 8 cm^3^) for experiments on inelastic neutron scattering (see references to our works in [5]). Part of this boule was used to prepare samples for experiments on inelastic light scattering (see references to our works in [5]). PMN samples from this boule were tested before experiments on neutron scattering, including at single-crystal neutron diffraction facilities in PSI, Villigen, Switzerland. It was shown that there are no deviations from the stoichiometry, composition, and structure of perovskite; the mosaic is less than 20′. From a boule, a sample in the form of a parallelepiped oriented as [001] with sizes 1 × 3 × 5 mm was prepared. The sample was oriented by using single-crystal X-ray diffraction.

A high-contrast 3 + 3 pass tandem Fabry–Perot interferometer combined with an optical microscope (Olympus BH-2) was used in the micro-Brillouin experiments. Scattering was excited by a green YAG laser with a wavelength of *λ* = 532 nm and power of 100 mW. A backscattering geometry, with a vertically polarized incident light and either a vertically or horizontally polarized scattered light, was employed. The phonon wave vector (**q**_ph_) was oriented along the [100] axis. The sample was put in a heat/cooling cell (THMS 6000) with temperature variations from −190 to 600 °C and stability of ±0.1 °C. When scanning the low-frequency region of the vibrational spectrum, several free spectral ranges (FSRs) of 6, 12, 75, 200, and 800 GHz were used. All observed spectra were polarized. To obtain the combined spectrum of Brillouin light scattering in PMN over a wide frequency range (from 0.6 to 800 GHz), the experimental Brillouin spectra obtained at different FSRs were merged together. The normalization was carried out for the spectrum obtained at FSR = 800 GHz. The measurements were carried out when the sample was cooled from 750 to 80 K at each of the FSRs indicated above. In the used geometry of the scattering, a longitudinal acoustic phonon was observed, the velocity of which is determined by the C_11_ elastic stiffness constant. The temperature behavior of this phonon is discussed in [26].

## 3. Results and Discussion

### 3.1. Broadband Brillouin Spectra in PMN

**Figure 1:**
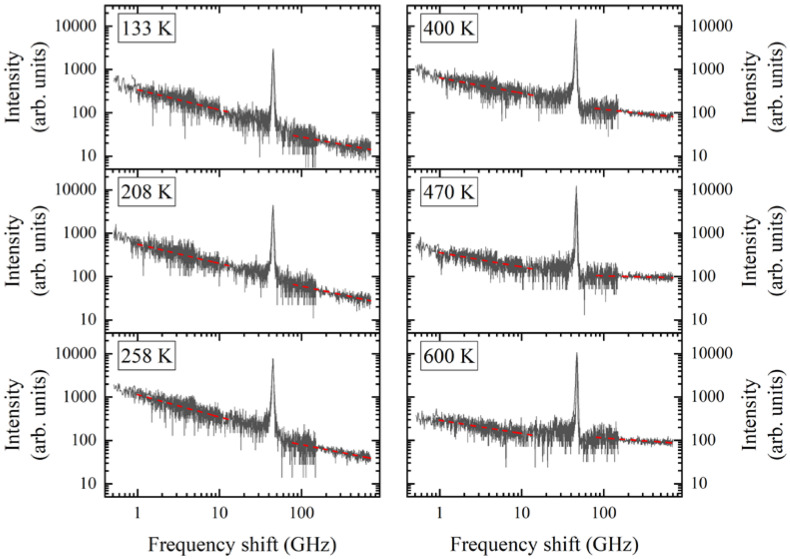
Combined spectra of Brillouin scattering in a PMN crystal obtained by “merging” the experimental spectra with different free spectral intervals (see text). Red dashed line is the result of a linear approximation.

Figure 1 shows the combined Brillouin scattering spectra, which reflect the evolution of the low-frequency region of the vibrational spectrum of the PMN crystal with temperature. The graph was plotted with the use of a double logarithmic scale for the convenience of describing QELS, the existence of which in PMN crystals is known from previous works [7,11]. Koreeda et al. [11] showed that QELS in the combined light-scattering spectra of PMN is well described by a power-law function (which is a superposition of a set of Lorentzians) centered at a zero frequency. On a double logarithmic scale, the power-law function is described as the linear dependence of intensity on frequency. The set of Lorentzians in QELS corresponds to the distribution of the relaxation times [11]. It is clearly seen from Figure 1 that the combined spectrum of inelastic light scattering consists of at least two linear sections corresponding to two QELS components: a narrow, low-frequency component in the range from 0.9 to 11 GHz and a wide, high-frequency component from 80 to 600 GHz. The high-frequency component continues into the THz range, being part of the QELS in the Raman spectra of PMN [7]. It is clearly seen that the spectra presented in Figure 1 cannot be described using the expression $I∼e\nu^{\alpha }$, as was conducted in [11]. An analysis of the intensity distribution of the spectra showed that the narrow and wide components of QELS can be described by two linear dependencies that correspond to two power-laws with different values for the exponents *α*–*α_N_* and *α_B_*, corresponding to the narrow and wide components, respectively. This indicates the existence of two relaxation time distribution functions in the considered frequency range and is consistent with the bimodal nature of the relaxation time distribution function obtained by analyzing the dielectric dispersion in PMN [27]. At a temperature of 285 K, we obtained the values α_N_ ≈ −0.5 and α_B_ ≈ −0.32. The differences in the combined spectra obtained and shown in [11] are apparently associated with different experimental conditions. Koreeda et al. [11] performed measurements using PMN with a horizontally polarized light incident along the [110] crystallography direction of the crystal and vertically polarized scattered light, whereas, in our experiments, a vertically polarized incident and scattered light along the [100] crystallography direction were used. In this case, apparently, various mechanisms which determine the distribution function of relaxation times manifest.

The intermediate region of the combined spectrum that separates the two QELS components (between 11 and 80 GHz) is highlighted since it contains a longitudinal acoustic phonon (LA) and an additional intensity value, which, in our opinion, is not related to either the wide or narrow QELS components (Figure 1). Let us consider this region of the combined spectrum and its temperature evolution (Figure 1). Figure 1, which was obtained at a temperature of 600 K, clearly shows that the linear function describing the broad QELS component is replaced by an intensity minimum in the vicinity of 60 GHz. This minimum passes into the asymmetric contour of the LA phonon. In the low-frequency region of the considered part of the spectrum, the excess intensity turns into a linear dependence for the intensity of the narrow QELS component. It should be noted that the shape of the LA phonon line is not described by the Lorentz function or by a damped harmonic oscillator. The characteristic distortion of the LA phonon line shape and the “dip” in the intensity of the high-frequency phonon wing in the vicinity of 60 GHz corresponds to the appearance of Fano resonance [28,29]. In our case, the Fano resonance effect means the existence of an interaction between the LA phonon and some broadband excitation, the frequency of which is comparable to but is less than the frequency of the LA phonon. The excess intensity, which apparently indicates the excitation interacting with the LA phonon, is weak and extended over a wide frequency range (about 80 GHz). The nature of this additional contribution to the combined spectrum in the vicinity of 60 GHz is not clear. Perhaps this is a manifestation of a soft overdamped optical mode, the existence of which is indicated by the results of experiments using neutron, hyper-Raman, and IR spectroscopy [5,9,30]. The only thing that does not match this assumption is the frequency of this excitation (<49 GHz), which is less than the experimentally obtained values of the soft optical mode frequency. Unfortunately, it is not possible to carry out the calculations, describe the shape of the phonon line, or determine the coupling constant in the framework of the Fano model due to the weak excitation intensity, which manifests itself as an excess intensity in the considered range of the combined spectrum. If this might be a soft, overdamped optical mode, then it apparently interacts with an acoustic phonon. It should be noted that the interaction of QELS and LA phonons can also take place. Lowering the temperature below 400 K changes the picture in the frequency region considered for the combined scattering spectrum; it is no longer possible to distinguish an additional intensity in the vicinity of 60 GHz, and the asymmetry of the LA phonon line contour, including the intensity minimum near the high-frequency region of the phonon line, disappears (the line shape is well described by the Lorentzian function) (Figure 1). Additional experiments on other scattering geometries and other crystallographic directions should clarify the reason for the distortion of the LA phonon line.

The study of the possible relaxation processes in PMN that are associated with the narrow and wide QELS components will be considered in the next section when analyzing the temperature behavior of the Brillouin spectra. It should be noted, again, that the attempt to build a combined spectrum for VH polarization was unsuccessful since the intensities of these spectra with different FSRs are close to the background level. This greatly distinguishes PMN from the other examples of the studied relaxors, in which QELS was observed in the VH polarization [8,12,13].

### 3.2. Narrow and Broad Components of QELS in Brillouin Spectra

Let us now return to the analysis of the narrow and broad QELS components in the PMN scattering spectra. These QELS components in the combined spectra most closely correspond to the experimental spectra of Brillouin light scattering with FSR = 12 GHz and 800 GHz. An analysis of these spectra with varying temperatures makes it possible to study the temperature evolution of the narrow and broad QELS components in PMN in detail. The quasielastic component contour is well described by a Lorentzian function centered at zero frequency, and the excitation is, thus, a Debye relaxator. By measuring the half-width at the half-height δν of the Quasielastic component, we can estimate the relaxation time, *τ*, as

(1) $$\tau =\frac{1}{2\pi \delta \nu }$$

According to the fluctuation-dissipation theorem, the spectral function S(ω) is proportional to the imaginary part of the susceptibility *χ″*(*ω*)

(2) $$S(\omega )\approx \frac{k_{b}T}{\hbar \omega }\chi \prime \prime (\omega )\;\mathrm{for}\;\hbar \omega <<k_{B}T$$

(3) $$\frac{T}{I_{CP}}\propto {\left(\int_{0}^{\infty }\frac{\chi \prime \prime (\omega )}{\omega }d\omega \right)}^{-1}\propto \chi \prime {\left(0\right)}^{-1}$$

where $k_{B}$ is the Boltzmann constant, and *T* is the temperature in Kelvins. Thus, the integral intensity of the quasielastic scattering, *I*_CP_, in the Brillouin spectra is proportional to the real part of the static susceptibility χ′(0). Thus, the determination of the experimental Brillouin spectra of the integrated intensity and half-width at half-height QELS (by fitting) makes it possible to obtain (expressions 1 and 3) the temperature dependencies of the susceptibility and the relaxation time of the narrow and wide QELS components.

**Figure 2:**
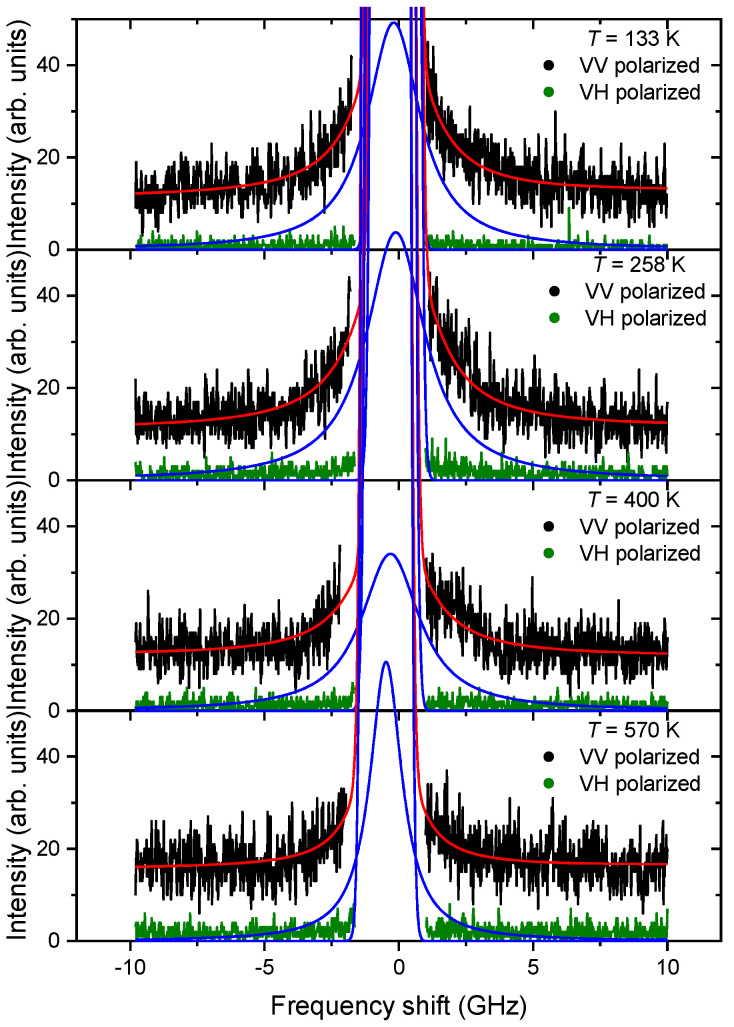
Polarized spectra of Brillouin light scattering in PMN (FSR = 12 GHz) and examples of their processing at different temperatures. The solid red line is the result of calculations using expression (4); fitting was carried out for the VV polarized spectra. Blue line is the contour of the QELS obtained as a result of data fitting (see text).

Let us consider the behavior of the Brillouin scattering spectra with FSR = 12 GHz. Figure 2 shows examples of the polarized spectra at various temperatures. The LA phonon is absent in the spectra with VV polarization since its frequency ω ≈ 49 GHz lies outside the existing frequency window. In Figure 2, for the VV polarization, one can clearly see the quasielastic scattering component centered at zero frequency, which is practically absent in the VH polarization. This suggests that the considered QELS component is polarized. It is well demonstrated in Figure 2 that QELS changes with temperature. The fitting of the experimental spectra, the examples of which are shown in Figure 2, was carried out using the sum of the Gaussian function describing the instrumental function, the Lorentzian function describing QELS, and the background intensity represented by the constant.

(4) $$I=A_{Gauss}\exp\left(-\frac{\omega_{Gauss}{}^{2}}{2\Gamma_{Gauss}{}^{2}}\right)+\frac{2A_{QELS}}{\pi }\frac{\Gamma_{QELS}}{4\omega^{2}+\Gamma_{QELS}{}^{2}}+I_{B}$$

**Figure 3:**
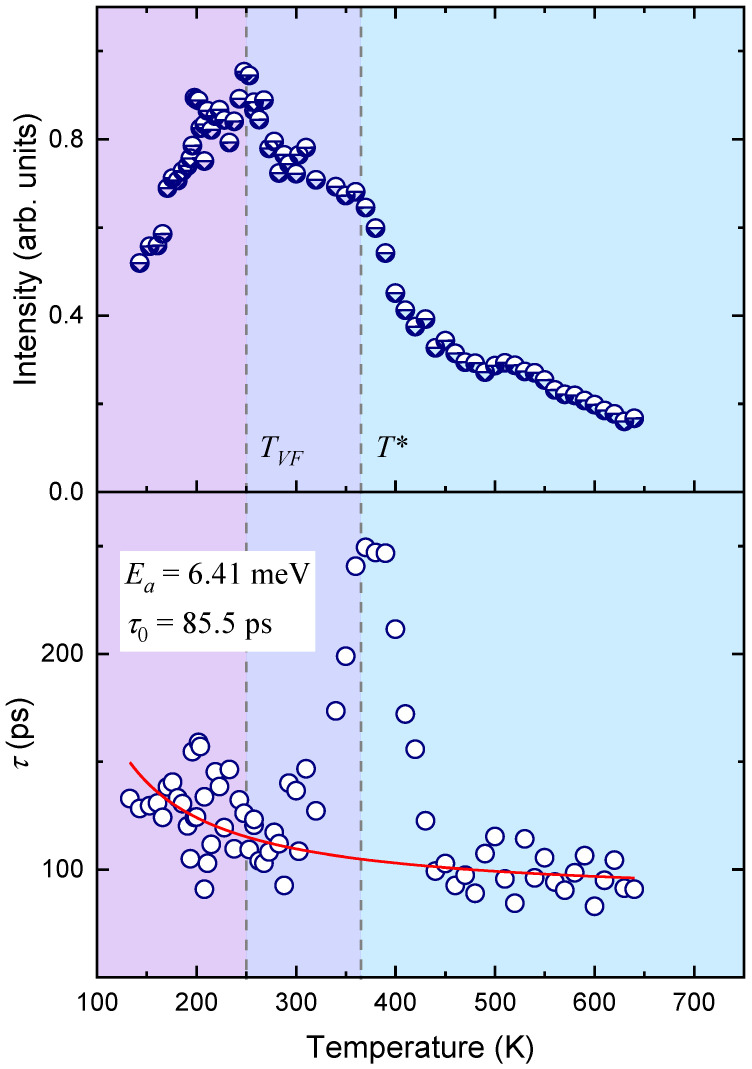
The temperature dependencies of intensity and relaxation time from the narrow QELS component in PMN. The circles are the results of calculations of experimental spectra with FSR = 12 GHz using Equations (1)–(4) (see text). The solid red line corresponds to the Arrhenius law. The colors in the figure highlight different temperature regions.

As a result of the calculations, we obtained the temperature dependencies of the integral intensity of the corresponding QELS component and the relaxation time (Figure 3). The intensity of the QELS in PMN in the Brillouin spectra with FSR = 12 GHz increases upon cooling, and a broad maximum with a complex structure is observed in a temperature range from 450 to 200 K. In this case, the intensity value increases by a factor of five in the vicinity of 250 K relative to its high-temperature values. Figure 3 shows that the maximum temperature dependence for intensity consists of two components: the first being a weak maximum in the region of 380 K, with the second being intense at 250 K, masking the first. At *T* < 250 K, the intensity of the QELS decreases. The temperature dependence of the relaxation time (*τ_s_*) demonstrates a weak temperature dependence during cooling, against the background of which the anomaly in the vicinity of 380 K is clearly visible. In this case, the value of *τ_s_* changes by a factor of 2.5 from 100 ps to 250 ps at *T* ≈ 380 K. The maximum in the observed anomaly *τ_s_* corresponds to a “shoulder” in the temperature dependence of the intensity and may reflect the critical dynamics of the corresponding relaxation process. The background change in relaxation time can be described by using the Arrhenius law $\tau_{s}=\tau_{0}\exp\left(E_{a}/k_{B}T\right)$ with the activation energy *E_a_* = 6.41 meV, and *τ*_0_ = 85.5 ps. These values do not agree with the values for the activation energy and the pre-exponential factor known to us from the literature and determined in the framework of the existing models. Therefore, we could not choose an appropriate relaxation mechanism. The anomalous behavior of the narrow QELS component in the vicinity of 380 K may be due to the interaction of the polarization fluctuations with a soft transverse optical mode. This is possible provided that the order parameter does not have a unique wave vector and is made up of a range of wave vectors around the center of the Brillouin zone, as it is assumed in [31]. Then, the occurrence of the interplay of the soft mode with the polarization fluctuations and the manifestation of the anomalous behavior of QELS in the vicinity of T* looks reasonable.

**Figure 4:**
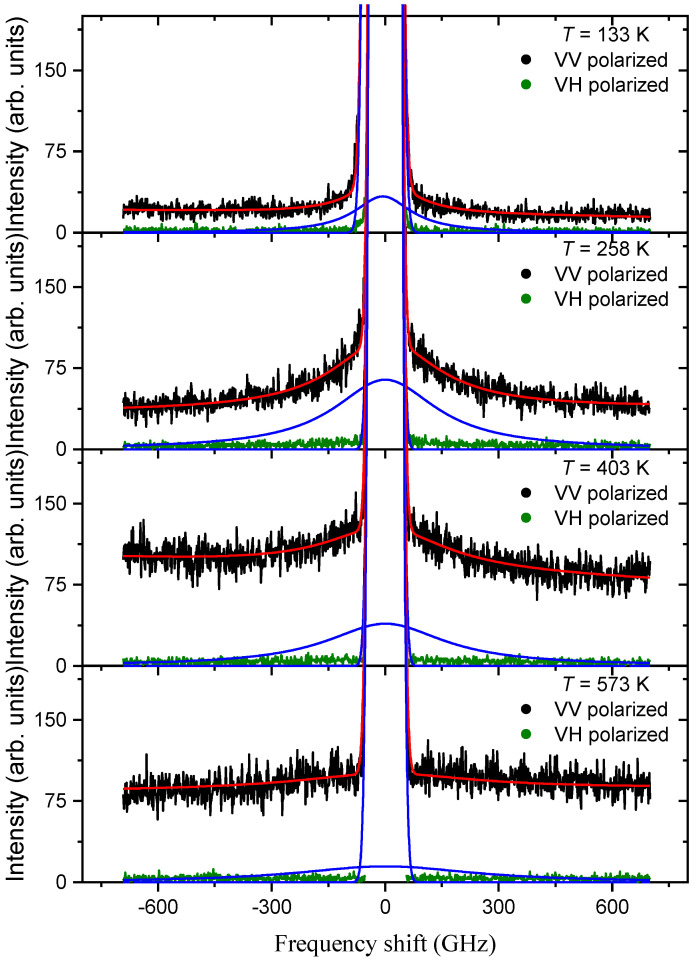
Polarized spectra of Brillouin light scattering in PMN (FSR = 800 GHz) and examples of their processing at different temperatures. The solid red line is the result of calculations using expression (4); fitting was carried out for the VV polarized spectra. Blue line is the contour of the QELS obtained as a result of data fitting (see text).

The behavior of Brillouin light scattering spectra in PMN with FSR = 800 GHz is shown in Figure 4. It reflects the temperature evolution of the broad QELS component. The scattering spectra, as can be seen in Figure 4, are polarized, and their changes in the VV polarization are clearly visible with decreasing temperature. The longitudinal acoustic phonon is not observed in the spectra because the width of the intense elastic scattering at a given FSR is comparable to the phonon frequency. The Brillouin spectra with FSR = 800 GHz (Figure 4) clearly show that, at high temperatures, the QELS is very wide, and its intensity is low. In Figure 1, it shows itself as an almost horizontal part of the high-frequency region of the combined spectrum, which increases slope angle with decreasing temperature. The experimental spectra in Figure 4 show that a decrease in temperature leads to an increase in intensity and a decrease in width.

**Figure 5:**
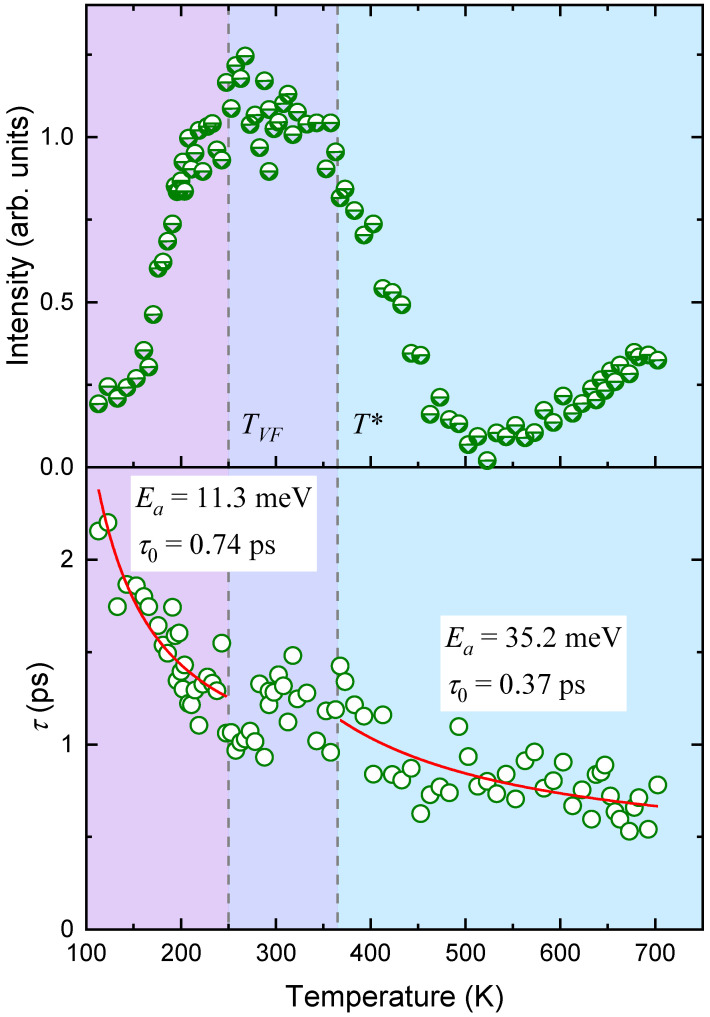
Temperature behavior of the intensity and relaxation time of the broad QELS component in PMN Brillouin spectra with FSR = 800 GHz. The circles are the results of fitting the experimental spectra by Equations (1)–(4); the line corresponds to the approximation of the data using the Arrhenius law. The colors in the figure highlight different temperature regions.

The results of fitting the experimental scattering spectra with FSR = 800 GHz are shown in Figure 5. It is clearly seen that the intensity of the QELS changes dramatically with decreasing temperature: a rather intense and broad component in the vicinity of 700 K decreases to minimum values in the region of 550 K and then begins to grow, increasing by more than an order of magnitude. The changes in intensity in the high-temperature region can be associated with the contribution of the soft optical mode. A broad anomaly in the temperature dependence of intensity, starting from 500 K, has a complex shape against the background of a maximum in the vicinity of 250 K, and a “shoulder” in the vicinity of 380 K can be distinguished. This intensity behavior correlates well with what we observe for the narrow QELS component (compare Figure 3 and Figure 5). Below 250 K, the QELS intensity drops, changing its value by less than a quarter of the maximum intensity. Apparently, the wide and structured intensity maximum reflects the existence of several processes that contribute to its anomalous behavior. The relaxation time (*τ_f_*) obtained from the broad QELS component of the Brillouin spectra measured with FSR = 800 GHz differs by two orders of magnitude from the relaxation time of the narrow component *τ_s_*. In the vicinity of room temperature, *τ_f_* ≈ 1 ps, and *τ_s_* ≈ 100 ps. A change in temperature leads to an increase in τ_f_, and this increase is not monotonous. It is necessary to highlight the temperature range between T* and *T_VF_*, where *τ_f_* is practically independent of temperature. We tried to describe *τ_f_(T)* in the temperature ranges T > T* and *T < T_VF_* using the Arrhenius law. At T > T*, the temperature dependence of *τ_f_(T)* is well described by the activation energy *E_a_* = 35.2 meV and *τ*_0_ = 0.3 ps, and at temperatures below *T_VF_*, *E_a_* = 11.3 meV and *τ*_0_ = 0.74 ps. These values do not agree with the values of *E_a_* and *τ*_0_ known to us from the literature for the cluster-flipping mechanism in PMN [30]. Studies on the behavior of longitudinal and transverse polarization fluctuations of the PNRs in QELS in PMN-56% PT [13] made it possible to determine the relaxation time and activation energy in the paraelectric phase. The relaxation time and activation energy of the longitudinal polarization fluctuations in PMN-56PT in the paraphase (*E_a_* = 26 meV and *τ*_0_ = 0.55 ps) are in accordance with the values, which were obtained during the characterization of the thermally activated changes in the relaxation time *τ_f_* of PMN at *T* > T*.

The dynamics of the PNRs at temperatures *T* < *T*_VF_, according to the results of studies on the dielectric response, are mainly determined by the vibrations of the walls of these regions, and the contribution of the polarization fluctuations from the PNR reorientation motion disappears. If we assume that this model is correct, then the behavior of *τ_f_*(*T*) in the considered temperature range is determined by the relaxation of the PNR walls. In the intermediate temperature range *T*_VF_ < *T* < T*, the behavior of *τ_f_*(*T*) is difficult to analyze due to the large scatter of points.

## 4. Conclusions

When summarizing the obtained results, it can be stated that in the Brillouin scattering of light in PMN within a frequency range from 0.7 GHz to 600 GHz, two-component quasielastic light scattering is observed. The narrow component is observed in the frequency range from 0.9 GHz to 11 GHz, and the broad component is observed in the frequency range from 80 GHz to 600 GHz. The intensity distribution in each of the components is described by a power law:$\;I∼e\nu^{\alpha }$ with different exponents. These parts of the combined Brillouin spectra correspond to either the different distribution functions of relaxation time or a bimodal distribution function of relaxation time. In the intermediate frequency range between 11 GHz and 80 GHz, a LA phonon is visible, the line shape of which depends on temperature. At T* < *T* < *T*_B_, the shape of the LA phonon line is distorted, and a noticeable “dip” is also observed on the high-frequency side of the line profile. Below T*, the line shape of the LA phonon is described by the Lorentz function. Similar distortions of the line shape are observed at the Fano resonance and are associated with the interaction of an acoustic phonon with a low-lying excitation.

The temperature behavior of the narrow and broad components of the QELS in PMN was analyzed based on the measured Brillouin spectra, with two specific frequency windows being selected, i.e., FSR = 12 GHz and 800 GHz, where different power-law behaviors for intensity were observed. Fitting the experimental spectra with the indicated free spectral intervals made it possible to plot the temperature dependencies of integral intensity and relaxation time. It was shown that the integrated intensity of both components behaves in a similar way, demonstrating a wide anomaly with two overlapping maxima in the vicinity of T* and *T*_VF_. The relaxation times of the broad and narrow components determined in the calculations differ by two orders of magnitude and are 1 ps and 100 ps, respectively (in the vicinity of room temperature). The behavior of the broad component with temperature change is different in different temperature ranges: at *T* > T*, the relaxation time *τ_f_*(*T*) is well described by the Arrhenius law and, apparently, is determined by longitudinal polarization fluctuations; at *T* < *T*_VF_, the behavior of *τ_f_*(*T*) is also thermally activated, with different activation energy. The temperature dependence of the relaxation time of the narrow component QELS *τ_s_*(*T*) is determined by two mechanisms: a thermally activated process with deceleration at low temperatures and critical deceleration with a maximum in the vicinity of T*. We cannot compare the activation energy and the characteristic attempted time *τ*_0_, which determine the behavior of the relaxation time in the frequency range from 0.9 to 11 GHz, with the data available in the literature since they are absent.

Special attention should be paid to the anomalous behavior of the Brillouin spectra of PMN in the vicinity of T*. Indeed, in the vicinity of 380 K, a “masked” maximum in the temperature behavior of the integrated intensity of the narrow and broad components of QELS can be observed, along with the critical behavior of the relaxation time *τ_s_*(*T*). A possible explanation for these anomalies might be an additional narrow component in QELS, which appears in the vicinity of 400 K. This assumption is consistent with the results of studies strictly into the elastic scattering in inelastic neutron scattering [5,24,32], where an increase in the intensity of this scattering is observed in the considered temperature range. In the present experiments, the width of this central peak is beyond our resolution.

Another important result of this study can be mentioned here. In the Brillouin scattering studies of partially disordered crystals, it is necessary to analyze the combined scattering spectra with different frequency ranges. This makes it possible to focus on the areas of study which are going to be investigated further.

## Data Availability

Not applicable.

## References

[1] Fleury P.A., Lyons K.B., Cummins H.Z., Levanyuk A.P. (1983). Central peaks near structural phase transitions. Modern Problems in Condensed Matter Sciences.

[2] Dasgupta R., Ballabh T.K., Tarafdar S. (1995). Quasielastic incoherent scattering in fractal systems. Phys. Rev. B.

[3] Wiedersich J., Adichtchev S.V., Rössler E. (2000). Spectral Shape of Relaxations in Silica Glass. Phys. Rev. Lett..

[4] Kojima S. (2022). 100th Anniversary of Brillouin Scattering: Impact on Materials Science. Materials.

[5] Cowley R.A., Gvasaliya S.N., Lushnikov S.G., Roessli B., Rotaru G.M. (2011). Relaxing with relaxors: A review of relaxor ferroelectrics. Adv. Phys..

[6] Cros L.E. (1987). Relaxor ferroelectrics. Ferroelectrics.

[7] Siny I.G., Lushnikov S.G., Katiyar R.S., Rogacheva E.A. (1997). Central peak in light scattering from the relaxor ferroelectric PbMg_1/3_Nb_2/3_O_3_. Phys. Rev. B.

[8] Kojima S., Ahart M., Sivasubramanian V., Bokov A.A., Ye Z.-G. (2012). Precursor dynamics of Pb(B_1/2_B’_1/2_)O_3_-type relaxor ferroelectrics studied by broadband micro-brillouin scattering. J. Adv. Diel..

[9] Kamba S. (2021). Soft-mode spectroscopy of ferroelectrics and multiferroics: A review. APL Mater..

[10] Lushnikov S.G., Jiang F.M., Kojima S. (2002). Central peak in the vibrational spectrum of the relaxor ferroelectric lead scandotantalate. Solid State Commun..

[11] Koreeda A., Taniguchi H., Saikan S., Itoh M. (2012). Fractal dynamics in a single crystal of a relaxor ferroelectric. Phys. Rev. Lett..

[12] Tsukada S., Kojima S. (2008). Broadband light scattering of two relaxation processes in relaxor ferroelectric 0.93Pb(Zn_1/3_Nb_2/3_)O_3_-0.07PbTiO_3_ single crystals. Phys. Rev. B.

[13] Helal M., Aftabuzzaman M., Tsukada S., Kojima S. (2017). Role of polar nanoregions with weak random fields in Pb-based perovskite ferroelectrics. Sci. Rep..

[14] Bokov A.A., Ye Z.-G. (2012). Dielectric relaxation in relaxor ferroelectrics. J. Adv. Diel..

[15] Hlinka J. (2012). Do we need the ether of polar nanoregions?. J. Adv. Diel..

[16] Eremenko M., Krayzman V., Bosak APlayford H.Y., Chapman K.W., Woicik J.C., Ravel B., Levin I. (2019). Local atomic order and hierarchical polar nanoregions in a classical relaxor ferroelectric. Nat. Commun..

[17] Fedoseev A.I., Popova E.A., Syrnikov P.P., Kojima S., Lushnikov S.G. (2015). Multi-component quasi-elastic light scattering in Na_1/2_Bi_1/2_TiO_3_ as studied by broadband Brillouin scattering. JETP Lett..

[18] Gvasaliya S.N., Lushnikov S.G., Sashin I.L., Siny I.G. (1999). Fractons in vibrational spectrum of the PbMg_1/3_Nb_2/3_O_3_ relaxor ferroelectric. Cryst. Rep..

[19] Gvasaliya S.N., Lushnikov S.G., Moriya Y., Kawaji H., Atake T., Smirnov M.B., Kazimirov V.Y. (2004). Specific Heat of Cubic Relaxor Ferroelectrics. J. Phys. Condens. Matter..

[20] Nuzhnyy D., Petzelt J., Bovtun V., Kempa M., Kamba S., Hlinka J. (2017). Infrared, terahertz, and microwave spectroscopy of the soft and central modes in Pb(Mg_1/3_Nb_2/3_)O_3_. Phys. Rev. B.

[21] Mathan N., Husson E., Calvarn G., Gavarri J.R., Hewat A.W., Morell A. (1991). A structural model for the relaxor Pb(Mg_1/3_Nb_2/3_)O_3_ at 5 K. J. Phys. Cond. Matter..

[22] Siny I.G., Smirnova T.A. (1989). Preceding paraphases in “diffuse transition” ferroelectrics. Ferroelectrics.

[23] Burns G., Scott B.A. (1973). Index of refraction in ‘dirty’ displacive ferroelectrics. Solid State Commun..

[24] Gvasaliya S.N., Roessli B., Cowley R.A., Huber P., Lushnikov S.G. (2005). Quasi-elastic scattering, random fields and phonon-coupling effects in PbMg_1/3_Nb_2/3_O_3_. J. Phys. Condens. Matter.

[25] Dkhil B., Gemeiner P., Al-Barakaty A., Bellaiche L., Dul’kin E., Mojaev E., Roth M. (2009). Intermediate temperature scale *T** in lead-based relaxor systems. Phys. Rev. B.

[26] Lushnikov S.G., Fedoseev A.I., Gvasaliya S.N., Kojima S. (2008). Anomalous dispersion of the elastic constants at the phase transformation of the PbMg_1/3_Nb_2/3_O_3_ relaxor ferroelectrics. Phys. Rev. B.

[27] Grigalaitis R., Banys J., Kania A., Slodczyk A. (2005). Distribution of relaxation times in PMN single crystal. J. Phys. IV France.

[28] Struzhkin V.V., Goncharov A.F., Hemley R.J., Mao H.-K. (1997). Cascading Fermi resonances and the soft mode in dense ice. Phys. Rev. Lett..

[29] Rahaman M.M., TImai T., Sakamoto T., Tsukada S., Kojima S. (2016). Fano resonance of Li-doped KTa_1−*x*_Nb*_x_*O_3_ single crystals studied by Raman scattering. Sci. Rep..

[30] Bovtun V., Kamba S., Pashkin A., Savinov M., Samoukhina P., Petzelt J., Bykov I.P., Glinchuk M.D. (2004). Central-peak components and polar soft mode in relaxor PbMg_1/3_Nb_2/3_O_3_ crystals. Ferroelectrics.

[31] Cowley R.A. (2012). Soft modes and structural phase transitions. Integr. Ferroelectr. Int. J..

[32] Gehring P.M., Hiraka H., Stock C., Lee S.-H., Chen W., Ye Z.-G., Vakhrushev S.B., Chowdhuri Z. (2009). Reassessment of the Burns temperature and its relationship to the diffuse scattering, lattice dynamics, and thermal expansion in relaxor Pb(Mg_1/3_Nb_2/3_)O_3_. Phys. Rev. B.

